# Inverted U-Shaped Relationship between Central Venous Pressure and Intra-Abdominal Pressure in the Early Phase of Severe Acute Pancreatitis: A Retrospective Study

**DOI:** 10.1371/journal.pone.0128493

**Published:** 2015-06-08

**Authors:** Chong Yang, Zhiyong Yang, Xinglin Chen, Tao Liu, Shanmiao Gou, Changzhong Chen, Jun Xiao, Xin Jin, Zhiqiang He, Liming Dong, Yushun Zhang, Na Luo, Heshui Wu, Chunyou Wang

**Affiliations:** 1 Pancreatic Disease Institute, Union Hospital, Tongji Medical College, Huazhong University of Science and Technology, Wuhan, Hubei Province, People’s Republic of China; 2 Organ Transplant Center, Sichuan Academy of Medical Sciences and Sichuan Provincial People's Hospital, Chengdu, Sichuan Province, People’s Republic of China; 3 School of Medicine, University of Electronic Science and Technology of China, Chengdu, Sichuan Province, People’s Republic of China; 4 Key Laboratory of Geriatrics of Health Ministry, Department of Geriatrics, Union Hospital, Tongji Medical College, Huazhong University of Science and Technology, Wuhan, Hubei Province, People’s Republic of China; 5 Surgical Oncology-Abdominal Department, Cancer Center, Union Hospital, Tongji Medical College, Huazhong University of Science and Technology, Wuhan, Hubei Province, People’s Republic of China; 6 Microarray Core Facility, Dana-Farber Cancer Institute, Boston, Massachusetts, United States of America; 7 Center for Applied English Studies, the University of Hong Kong, Hong Kong, People’s Republic of China; University of Szeged, HUNGARY

## Abstract

**Objective:**

Many studies have indicated that intra-abdominal pressure (IAP) is positively correlated with central venous pressure (CVP) in severe cases. However, although elevated IAP is common in patients with severe acute pancreatitis (SAP), its relationship with CVP remains unclear. Our study aimed to investigate the association of IAP with CVP in early-phase SAP patients.

**Methods:**

In total, 116 SAP patients were included in this retrospective study. On the first day of hospitalization, blood samples were collected for biochemical examination and cytokine concentration monitoring. Additionally, a urinary catheter and right subclavian vein catheter were inserted for IAP and CVP measurement, respectively. Other routine clinical data were also recorded.

**Results:**

Within 24 hours after hospitalization, CVP fluctuated and increased with increasing IAP up to 15.7 mmHg (*P* = 0.054) but decreased with increasing IAP when the IAP was > 15.7 mmHg (*P* < 0.001). After adjusting for abdominal perfusion pressure (APP) and mean arterial pressure (MAP), a similar distribution was observed. An inverted U-shaped trend between IAP and CVP was also present in the groups classified according to the patient’s sex, local complications, ascites, and serum amylase levels.

**Conclusions:**

CVP and IAP have an inverted U-shaped relationship, with a peak at an IAP of 15.7 mmHg in the early phase of SAP. After this peak, CVP decreases as IAP increases. These results have crucial implications for clinical fluid resuscitation in SAP patients. In particular, because one CVP value might be correlated with different IAP values in patients with the same CVP, the volume of fluid needed might be different.

## Introduction

Acute pancreatitis (AP) is an acute inflammatory process with variable involvement of local tissues and remote organ systems [1]. Elevated intra-abdominal pressure (IAP) is common in the early phase of AP [2], particularly in patients with severe acute pancreatitis (SAP) [3,4]. Persistent intra-abdominal hypertension (IAH) can lead to complications, including diaphragmatic compression with reduced pulmonary compliance, renal dysfunction, and intestinal ischemia [5], all of which can increase mortality from AP.

IAH can also lead to cardiovascular dysfunction. Increased IAP directly compresses the mediastinum and inferior vena cava, inducing a low cardiac index [6]. Additionally, over-expression of inflammatory mediators, such as interleukin (IL)-6, IL-8, and tumor necrosis factor (TNF)-α, injure the endothelium of the microcirculatory system and subsequently increase capillary permeability, leading to capillary leakage syndrome [7]. At the same time, the increased capillary permeability leads to fluid loss from the intravascular space and fluid sequestration into the third space [8], aggravating the existing IAH. These synergistic effects result in redistribution of the blood circulation and hypovolemic status. Therefore, early fluid resuscitation is important for reducing morbidity in patients with SAP [9].

Currently, the theory that early goal-directed therapy (EGDT) reduces morbidity and mortality among patients with AP has been accepted worldwide [9–11]. Central venous pressure (CVP), which is the pressure recorded from the right atrium or superior vena cava, is commonly monitored and is frequently used as the major indicator in decisions regarding fluid administration. In particular, it has been found that CVP is positively correlated with IAP in both animals and humans [12–15]. However, whether there is a positive relationship between CVP and IAP in early-phase SAP patients with simultaneous IAH and inadequate circulatory blood volume is unknown.

Therefore, the aim of this study was to evaluate the relationship between IAP and CVP during the early phase of SAP.

## Materials and Methods

### Data source

All relevant clinical data were extracted from the database of a multi-center, randomized controlled trial (RCT) that was conducted from October 1, 2007, to November 31, 2008, as previously described [16]. The measurements, including IAP and CVP monitoring and routine clinical data collection, were performed based on default protocols. After excluding irrelevant data, a total of 116 SAP patients from four study sites were enrolled in this retrospective study.

### Patients

In this study, all of the participants were adult patients (aged 18 to 70 years old) admitted within 3 days of disease onset. The diagnostic and classification criteria for SAP were in accordance with the revised Atlanta criteria of 2013 [17], including two of the following features: (1) abdominal pain consistent with AP; (2) amylase activity at least three times greater than the upper limit of normal; and (3) characteristic findings of AP on contrast-enhanced computed tomography (CT) and, less commonly, on magnetic resonance imaging or transabdominal ultrasonography. Patients who had cardiac failure or pulmonary edema, were pregnant, underwent early surgical treatment, or presented with other factors that could affect CVP and IAP were excluded. All of the patients received specialized medical therapy for AP according to the United Kingdom, Chinese Medical Association, and International Association of Pancreatology guidelines [18–20]. Moreover, a urinary catheter and a central venous catheter were inserted for IAP and CVP measurement, respectively. In addition to its use in routine biochemical examination, blood serum was obtained for the analysis of inflammatory cytokines in all patients. This study was approved by the Medical Ethics Committee of Union Hospital (Wuhan, China). The need for informed consent was waived by the Medical Ethics Committee because the study was an observational, retrospective study using a database from which the patients’ identification information had been removed.

### IAP and CVP measurement protocols

1. IAP measurement and IAP grade classification were performed according to the standard techniques established by the World Society of Abdominal Compartment Syndrome in 2006 [21]. The IAP measurement protocol was as follows: a catheter was inserted into the bladder; 25 mL of 0.9% saline was instilled, and the midaxillary line was considered to be level 0. IAP was determined at 10:00, 12:00, and 14:00 on the first day after admission, and the mean IAP value was calculated as the mean level achieved among the measurements. The urinary catheter was removed when the patients were in comparatively stable condition or were suspected of having a urinary tract infection. We present the IAP (mmHg) values according to the following formula: 1 (cmH_2_O) = 0.76 (mmHg).
2. The CVP measurement protocol was as follows: a 7-F single-lumen catheter (Arrow International Inc., PA, USA) was inserted into the right subclavian vein, and the position of the catheter tip in the superior vena cava directly adjacent to the right atrium was confirmed using radiography, if necessary. The CVP catheter was removed when the patients were suspected of having a catheter tip infection. CVP and IAP were measured simultaneously, and the mean CVP was defined as the mean level achieved among all of the measurements.

### Data collection

The factors included in the present investigation were age, sex, cause of illness, IAP, CVP, mean arterial pressure (MAP), abdominal perfusion pressure (APP; APP = MAP−IAP), 24-hour urine output, and the number of fluid collections on CT (based on blinded radiologists' reports). APACHE II scores, serum amylase, white blood cell counts, glucose, and albumin were measured on the first day after admission. MAP and APP were monitored simultaneously with IAP and CVP. The fluid balance within the first 24 hours was also calculated. CT grade classification was assessed according to Balthazar’s methods [22]. Local complications, such as acute peripancreatic fluid collection, acute necrotic collection and pancreatic pseudocysts, were recorded. Serum high-sensitivity C-reactive protein (hs-CRP) and inflammatory cytokine density were measured in the Central Laboratory of Wuhan Union Hospital. Serum hs-CRP density were measured using a Cobas Integra 800 instrument (Roche, Basel, Switzerland). The serum densities of IL-1, IL-6, IL-8, and TNF-α were measured using enzyme-linked immunosorbent assays (R&D Systems, Minneapolis, MN, USA) according to the manufacturer’s instructions.

### Statistical analysis

Both univariate and multivariate logistic regression analyses were performed. CVP was measured in cmH_2_O. We first divided the patients into three groups according to their CVP values and then examined the inter-group differences with regard to the patients’ general conditions, liver and renal function, inflammatory cytokine density, IAP values, CVP values, CT grades, and APACHE II scores. The descriptive results of the analysis of normally distributed data are expressed as the means ± standard deviations, and medians (ranges) were evaluated using one-way ANOVA or the Kruskal test (rank-sum test). Chi-square analysis was also performed for categorical variables, and non-normally distributed values were evaluated using the Mann-Whitney U test.

We used a linear regression model to identify potential confounders and then created a scatter plot of CVP (cmH_2_O) versus IAP (mmHg). We also created a scatter-plot of CVP (cmH_2_O) versus IAP (mmHg) within different groups categorized according to the following variables: sex (male or female), local complications (no or yes), ascites (no or yes), and amylase density (low or high, according to the median value).

We then applied a generalized additive model using a spline smoothing function to examine the relationship between CVP and IAP, and we conducted piecewise linear regression analysis to fit the smoothing curve, with adjustment for potential confounders. The turning points connecting the piecewise lines were determined by trial and error, including the selection of turning points along a pre-defined interval and the choice of turning points that yielded the maximum likelihood model. The piecewise linear model was then tested using a log-likelihood ratio test against the one-line linear regression model.

Finally, we applied multiple regression analyses to estimate the correlation coefficient and 95% confidence interval (CI) between IAP and CVP, with adjustment for potential confounders. All of the analyses were performed using Empower(R) (www.empowerstats.com, X&Y Solutions, Inc., Boston, MA, USA) and R (http://www.R-project.org) software, as described by Yu *et al*. [23]. The missing data are described as "NA" as per Empower(R) requirements. A value of *P* < 0.05 was considered statistically significant.

## Results

**Fig 1:**
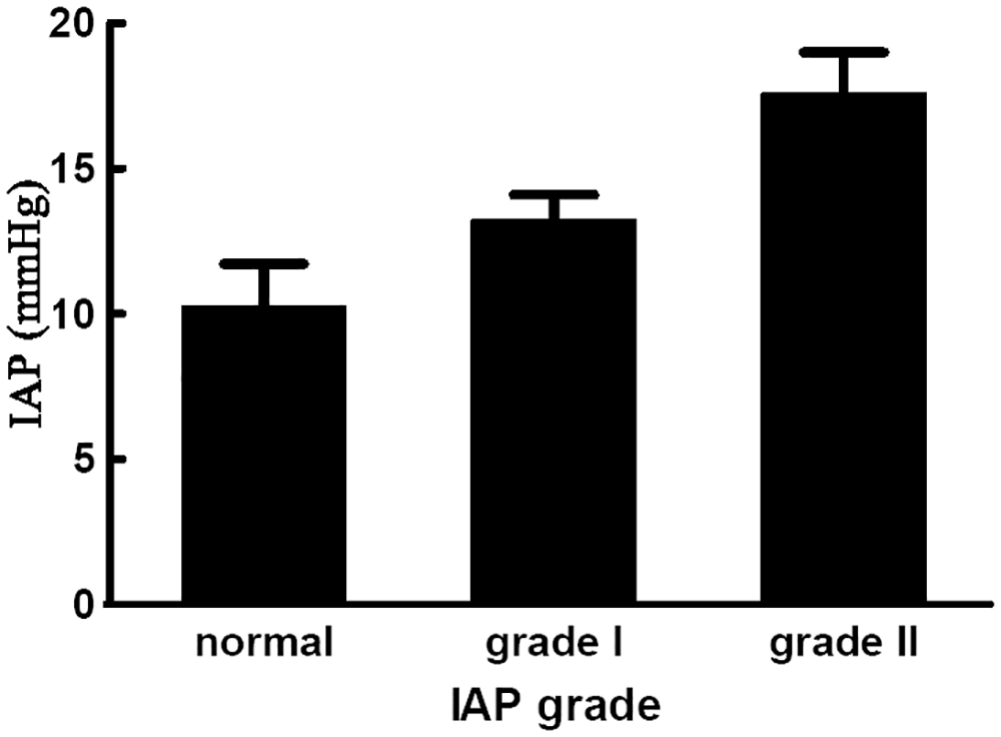
Bar chart representing the frequency of different grades of IAH according to the World Society for Abdominal Compartment syndrome guidelines. Normal: IAP<12mmHg; GradeI IAH: IAP between 12 to 15 mmHg. GradeII IAH: IAP between 16 to 20 mmHg. GradeIII IAH: IAP between 21 to 25 mmHg. GradeIV IAH: IAP greater than 25 mmHg. IAP: intra-abdominal pressure, IAH: intra-abdominal hypertension.

A total of 116 patients were included in the study. On the first day of admission, there were 76 (65.5%), 21 (18.1%) and 7 (6.03%) patients who suffered from respiratory, renal and cardiovascular failure, respectively. Fifty-eight (50.0%) developed persistent respiratory failure, and 9 (7.76%) developed persistent renal failure. According to the revised Atlanta definitions and classifications, 52 (44.8%) patients presented with moderate SAP and 64 (55.2%) patients presented with SAP. Sixty-nine (59.5%) patients presented with one or more local complications, and 15 (12.9%) patients presented with moderate to large ascites in the peritoneal cavity. The mean IAP, MAP, APP, and CVP of the patients in this study were 12.3 mmHg, 98.2 mmHg, 85.8 mmHg and 9.9 cmH_2_O, respectively. The mean age was 47.2 ± 11.9 years. The mean urine output during the first 24 hours after admission was 2642 ± 1624 mL. The mean serum alanine transaminase (ALT) and aspartate transaminase (AST) densities were 57.0 ± 83.1 U/L and 58.3 ± 95.4 U/L, respectively. The mean serum BUN and Cr values were 5.7 ± 2.7 mmol/L and 82.4 ± 35.1 μmol/L, respectively. Table 1 shows the characteristics of the study population according to CVP, and Fig 1 shows the distribution of the mean IAP.

**Table 1:** Characteristics of the study population by central venous pressure quartiles.

	Q1 (CVP <5)	Q2 (5≤CVP<12)	Q3 (CVP≥12)	*P* value	*P* value*
Patients, n	7	81	28		
Age, year	46.4 (16.8)	47.2 (12.0)	47.3 (10.4)	0.986	0.937
Gender				0.236	0.261
Male	6 (85.7%)	48 (59.3%)	20 (71.4%)		
Female	1 (14.3%)	33 (40.7%)	8 (28.6%		
Height, cm	170.6 (7.4)	166.5 (7.7)	165.7 (6.5)	0.3	0.226
BUN, mmol/L	5.1 (3.0)	5.5 (2.5)	6.4 (3.0)	0.23	0.247
Cr, μmol/L	74.2 (30.9)	78.7 (31.3)	95.0 (43.8)	0.087	0.197
ALT, U/L	33.7 (18.5)	54.8 (79.8)	69 (100.8)	0.556	0.643
AST, U/L	28.4 (14.7)	57.8 (102.5)	67.1 (85.1)	0.633	0.15
Fluid balance (24h), ml	1068.6 (758.2)	584.9 (1273.0)	509.1 (1234.0)	0.564	0.46
Urine, ml	2571.4 (1014.4)	2495.1 (1490.1)	3082.5 (2022.1)	0.254	0.377
BE, mmol/L	-4.6 (4.1)	-3.3 (4.7)	-4.6 (3.4)	0.359	0.059
SPO2, %	74.3 (24.7)	79.8 (24.0)	73.5 (24.1)	0.451	0.095
APACHE II score	23.9 (5.0)	12.5 (4.9)	11.1 (2.9)	<0.001	<0.001
Serum amylase, U/L	529.9 (345.6)	701.2 (599.3)	787.1 (667.7)	0.582	0.735
hs-CRP, μg/ml	190.3 (15.0)	176.9 (95.2)	240.7 (111.0)	0.019	0.037
IL-1, pg/ml	19.2 (5.9)	24.5 (15.8)	21.6 (13.6)	0.492	0.614
IL-6, pg/ml	29.8 (18.9)	52.1 (61.2)	67.0 (52.5)	0.265	0.032
IL-8, pg/ml	69.7 (84.4)	29.3 (27.6)	29.4 (20.6)	0.007	0.498
TNF-α, pg/ml	70.3 (65.1)	43.0 (19.4)	35.6 (13.1)	0.003	0.088
IAP, mmHg	18.4 (1.0)	11.8 (2.9)	12.3 (2.1)	<0.001	<0.001
CVP, cmH2O	3.6 (0.7)	9.2 (1.5)	13.5 (1.5)	<0.001	<0.001
MAP, mmHg	92.4 (8.4)	97.1 (10.5)	102.8 (11.4)	0.019	0.002
APP, mmHg	74.0 (7.6)	85.3 (10.7)	90.5 (11.6)	0.001	<0.001
HR, n/min	116.0 (9.9)	97.2 (17.0)	107.2 (17.3)	0.001	<0.001
RBC, /L	4.1 (0.8)	6.0 (11.0)	4.9 (0.8)	0.789	0.065
CT grade, score				0.037	0.033
3	7 (100%)	50 (62.5%)	11 (39.3%)		
4	0 (0%)	5 (6.2%)	4 (14.3%)		
5	0 (0%)	25 (31.2%)	13 (46.4%)		
Ascites				0.96	0.899
No	6 (85.7%)	71 (87.7%)	24 (85.7%)		
Yes	1 (14.3%)	10 (12.3%)	4 (14.3%)		
Local complication				0.06	0.077
No	1 (14.3%)	30 (37.0%)	16 (57.1%)		
Yes	6 (85.7%)	51 (63.0%)	12 (42.9%)		

CVP: central venous pressure; IAP: intra-abdominal pressure; MAP: mean arterial pressure; APP: abdominal perfusion pressure; HR: heart rate; Ascites: moderate-to-large fluid collections in the peritoneal cavity detected by B type ultrasonography or CT scans; APACHE II score: Acute Physiology and Chronic Health Evaluation II score; BE: base excess; CT grade: according to Balthazar's methods; Local complication: including pancreatic necrosis, pseudocyst and abscess.

*P* value*: Kruskal Wallis rank sum test was used to examine the significance if the variable was continuous. If theoretical number of the enumeration data <10, Fisher's exact test was used to calculate the significance.

[mean (SD) and median (min-max) or n(%)]

**Fig 2:**
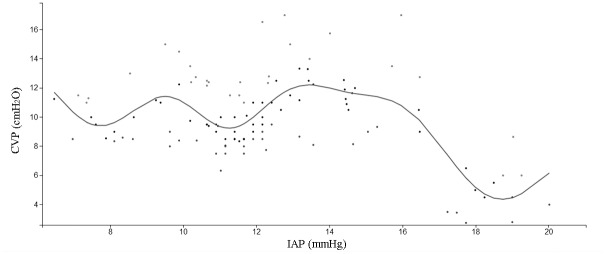
Scatter plot and smoothing curve of IAP (mmHg) by CVP (cmH_2_O).

Fig 2 is a scatter plot of CVP (cmH_2_O) versus IAP (mmHg). The curve shows that the relationship between CVP and IAP was not simply linear; rather, CVP fluctuated and increased with increasing IAP up to an IAP of approximately 16 mmHg. In particular, as shown in Table 2, threshold effect analysis indicated that CVP increased with increasing IAP up to an IAP of 15.7 mmHg. In patients with an IAP ≤ 15.7 mmHg, CVP was not significantly associated with IAP (β = 0.21, 95% CI: 0.00 to 0.40, *P* = 0.054). In patients with an IAP > 15.7 mmHg, the correlation coefficient (β) of CVP was negative (β = -2.12, 95% CI: −2.67 to −1.57, *P* < 0.001).

**Table 2:** Threshold effect analysis of IAP in CVP using piecewise linear regression model.

Model	Result[β (95%CI) *P* value]
Model I one-line linear regression model	-0.31 (-0.47, -0.15) <0.001
Model II turning point	15.7
group1: IAP ≤ 15.7, correlation coefficient (β1)	0.21 (0.00, 0.41) 0.054
group2: IAP > 15.7, correlation coefficient (β2)	-2.12 (-2.67, -1.57) <0.001
β2-β1	-2.32 (-3.01, -1.63) <0.001
a log likelihood ratio test	<0.001
predictive value of CVP at turning point	11.37 (10.4, 12.34)

Outcome variance: CVP (cmH2O); Risk factor: IAP (mmHg).

**Fig 3:**
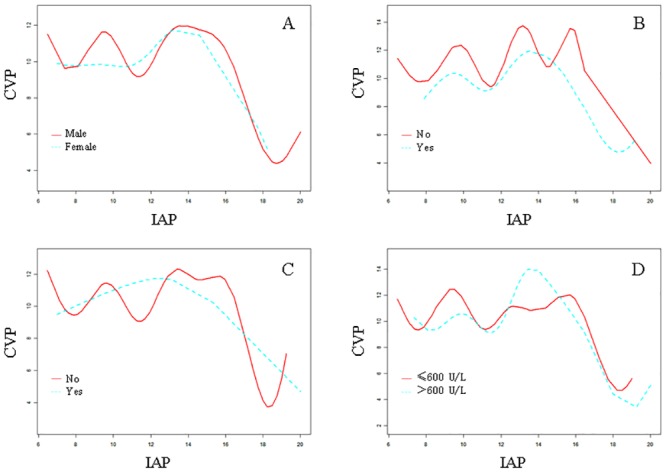
Smoothing curve of CVP (cmH_2_O) by IAP (mmHg) in different groups considering. A: patients' gender; B: local complication; C: ascites; D: serum amylase.

Table 3 shows the associations between CVP and IAP based on a multiple regression model adjusted for MAP and APP. CVP was marginally significantly positively associated with IAP (β = 0.20, 95% CI: 0.01 to 0.41, *P* = 0.059) in patients with an IAP ≤ 15.7 mmHg. However, in patients with an IAP > 15.7 mmHg, the correlation coefficient of CVP was negative (β = −2.14, 95% CI: −3.42 to −0.85, *P* = 0.005). This result demonstrated that CVP increased among patients with AP who had an IAP ≤ 15.7 mmHg. However, when the IAP of patients with AP was > 15.7 mmHg, the CVP decreased. After adjusting for APP and MAP, a similar distribution was still observed. In addition, an inverted U-shaped relationship between CVP and IAP was observed when the patients were classified according to sex, local complications, ascites, and serum amylase values (see Fig 3).

**Table 3:** Adjusted Effect of IAP on CVP [β (95%CI) *P* value].

	correlation coefficient (β)	95%CI	*P* value
IAP group 1 (IAP≤15.7)			
Model I	0.2	-0.01, 0.41	0.059
Model II	0.22	0.01, 0.42	0.042
Model III	0.18	-0.03, 0.39	0.094
IAP group 2 (IAP>15.7)			
Model I	-2.14	-3.42, -0.85	0.005
Model II	-1.9	-2.73, -1.06	<0.001
Model III	-2.128	-2.30, -1.294	<0.001

Outcome variance: CVP (cmH_2_O); risk factor: IAP (mmHg).

Model I: Multiple regression model (no adjusted related risk factors);

Model II: Multiple regression model adjusted APP level;

Model III: Multiple regression model adjusted MAP level.

CVP: central venous pressure; IAP: intra abdominal pressure; MAP: mean arterial pressure; APP: abdominal perfusion pressure.

Table 4 shows the characteristics of the study population according to IAP levels. Patients with IAP values >15.7 mmHg exhibited higher APACHE II scores (*P* < 0.001), higher hs-CRP levels (*P* = 0.011), more balanced fluid amounts (*P* = 0.018), and lower SPO_2_ levels (*P* = 0.033).

**Table 4:** Characteristics of the study population by intra-abdomnal pressure quartiles.

	Q1 (IAP≤15.7 mmHg)	Q2 (IAP>15.7 mmHg)	*P*
Patients, n	99	17	—
APACHE II score	11.7±3.7	19.8±7.6	<0.001
hs-CRP, μg/ml	184.7±101.7	235±74.5	0.011
Balance amount, mL	497±1275	1168±801	0.018
SPO2, %	79.5±23.6	68.8±25.1	0.033
CT grade (n,%)			0.090*
3	54 (55.1)	14 (82.4)	
4	9 (9.2)	0 (0.0)	
5	35 (35.7)	3 (17.6)	
TNF-α, pg/ml	41.2±18.8	52.7±43.6	0.255
APP, mmHg	99.2±18.5	113.7±11.1	0.002*
MAP, mmHg	98.7±11.1	96.9±11.8	0.513
Urine, mL	2671±1710	2442±883	0.572
RBC, /L	5.8±9.9	4.5±0.8	0.581

Q1: the patients with IAP≤15.7mmHg; Q2: the patients with IAP>15.7 mmHg.

*P*: value from Kruskal Test (rank sum test);

*P: value from ANOVA.

RBC: count of red blood cell; APACHE II score: Acute Physiology and Chronic Health Evaluation II score; APP: abdominal perfusion pressure; MAP: mean arterial pressure.

[mean±SD or n(%)]

## Discussion

Our study demonstrated that the relationship between CVP and IAP in the early phase of SAP was not a simple linear correlation. Further threshold effect analysis demonstrated that CVP and IAP had an inverted U-shaped relationship, with the peak IAP at 15.7 mmHg (approximately grade II IAH). After the peak value, CVP decreased as IAP increased. This relationship was marginally significant (β = 0.20, 95% CI: 0.00 to 0.40, *P* = 0.054). However, in patients with a higher IAP (IAP > 15.7 mmHg), the correlation between IAP and CVP was significantly negative (β = −2.12, 95% CI: −2.67 to −1.57, *P* < 0.001). A similar inverted U-shaped relationship between CVP and IAP was still found after adjusting for APP and MAP, and it persisted in the groups classified according to the patient’s sex, local complications, ascites, and serum amylase value.

CVP is frequently used for the assessment of cardiac preload and volume status [24]. Previously, many studies have documented a positive correlation between IAP and CVP [14,15,25]. For example, Kuntscher *et al*. reported that the CVP level was positively correlated with IAP in patients with major burns [26]. However, in the present study, we found that the relationship between IAP and CVP was not linear but rather formed an inverted U-shape. One reason for this difference was that our subjects were patients with AP who had more severe capillary permeation than other critical patients with the same IAP levels.

IAP, an early marker of AP severity, is closely correlated with capillary permeation in patients with SAP [3,27]. Moreover, several studies have indicated that IAH was associated with high rates of pancreatic infection, septic shock, organ dysfunction, and mortality in patients with SAP [12,28–30]. In our study, patients with IAP values >15.7 mmHg exhibited higher APACHE II scores (*P* < 0.001), higher hs-CRP levels (*P* = 0.011), more balanced fluid amounts (*P* = 0.018), and lower SPO_2_ levels (*P* = 0.033) (Table 4). As IAP increased, capillary permeability increasingly worsened, resulting in a lower blood volume. Once the “volume-decreasing effect” exceeded the “pressure-increasing effect”, CVP did not increase but rather decreased with increasing IAP.

Our study has potential implications for early fluid resuscitation in patients with SAP. CVP monitoring is widely used in clinical work for the assessment of cardiac preload and volume status [31]. Dellinger *et al*. stated that CVP should continue to be recommended to guide fluid resuscitation [32,33]. Additionally, Mayerle *et al*. recommended that CVP, combined with the urine excretion rate and daily hematocrit, should be closely monitored and that CVP should be increased to a level between 8 and 12 cmH_2_O for the management of AP patients [34]. Moreover, certain researchers have reported that the CVP could be used to gauge fluid balance within 12 hours after onset in septic shock patients [35]. Because of the effects of IAP, the monitored CVP value should be adjusted to the real value for fluid resuscitation. Most previous studies have reported that the CVP level was positively correlated with IAP in critical patients [1–4]. Based on this previously reported correlation between IAP and CVP, it was recommended that clinical practitioners adjust the real value for fluid resuscitation. However, our study indicated that there was an inverted U-shaped relationship between CVP and IAP in the early phase of AP, indicating that the same CVP level might be present in patients with SAP that have different levels of IAP and varying severity of disease. Accordingly, when adjusting the monitored CVP value to the real value for fluid resuscitation, both disease severity and increasing IAP should be considered. Thus, in the case of two patients with the same CVP, a larger volume of fluid is needed for resuscitation in the patient with the higher IAP level.

In recent years, the theory that CVP could be used to guide fluid therapy has been challenged. In fact, several studies have demonstrated that CVP did not reflect the body’s blood volume and, therefore, that CVP should not be used to make clinical decisions regarding fluid management [36–38]. In a recently published meta-analysis, Marik *et al*. specifically stated that CVP should not be used to make clinical decisions regarding fluid management because this parameter alone did not improve upon earlier methods that served as a guide for fluid resuscitation [39]. Our study also showed that CVP could remain the same at different IAP values, suggesting that CVP is unreliable as the sole indicator of the need for fluid management. However, we maintain that CVP should still be used as an important index for fluid resuscitation. Currently, the theory that EGDT is important for reducing morbidity and mortality among patients with AP is accepted worldwide [9–11]. In 2013, the acute pancreatitis management guidelines issued by the American College of Gastroenterology indicated that early and aggressive intravenous hydration was most beneficial within the first 12–24 hours of onset and might have little benefit for AP patients beyond this time [40]. Although the gold standard for fluid resuscitation in SAP patients remains unspecified, the criteria used worldwide for fluid resuscitation in patients with SAP are commonly combinations of indices, including factors such as pulse, blood pressure, urine output, CVP, and MAP [41,42]. In our previous RCT, the criteria for fluid resuscitation among patients with SAP were as follows: CVP, 8–15 mmHg; urine output, ≥ 0.5 mL·kg^−1^·h^−1^; MAP, ≥ 65 mmHg; heart rate, 80–110 bpm; hematocrit, ≥ 0.3; and venous oxygen saturation, ≥ 0.70 [16]. Clinically, we have also adopted the same indices, which have proved to be helpful in relieving symptoms. Therefore, we maintain that, although the CVP value alone is not sufficient to reflect the body's blood volume or even to guide fluid resuscitation, CVP combined with other indices remains important for guiding fluid resuscitation. Further studies are needed to develop a sound theory as groundwork for detailed guidelines for SAP patients.

There were certain limitations to our study. First, it was designed as a retrospective study and involved only a Chinese population. Further research is needed that includes other potential confounding factors that could influence the relationship between IAP and CVP in patients with early-phase AP, including differences in characteristics between Chinese populations and other populations, such as disease etiology and body mass index. Second, the number of patients included was limited (n = 116), and only 17 patients had an IAP level > 15.71 mmHg. Despite the trend toward CVP increasing and then decreasing with increasing IAP in different groups classified according to the patient’s sex and APACHE II scores, the associations between CVP and IAP became non-significant after adjusting for certain factors (*P* = 0.154, after adjusting for the hs-CRP level, local complications, and the IL-6 level). Future studies should include more patients, particularly more patients with higher IAP levels, to validate the relationship between IAP and CVP. Third, our study only included data from the first day of hospitalization in patients with SAP. Whether a similar relationship between IAP and CVP persists later also merits further investigation. The fourth limitation was that the specific reason for the inverted U-shaped relationship between CVP and IAP was not revealed in this observational study; therefore, future animal and clinical studies should be initiated to determine the mechanism underlying this relationship.

## Conclusions

This study suggested that CVP and IAP had an inverted U-shaped relationship, with the peak IAP at 15.7 mm Hg in early-phase SAP patients. After this peak, CVP decreased as IAP increased. An inverted U-shaped relationship between CVP and IAP was also observed after adjusting for APP and MAP, and this shape persisted in the different groups classified according to the patient’s sex, local complications, ascites, serum amylase, CT grades, and APACHE II scores. These results have crucial implications for clinical fluid resuscitation in SAP patients. In particular, because one CVP value might be correlated with different IAP values in SAP patients with the same CVP, the amount of fluid required might be different.

## Supporting Information

S1 Table Relevant data underlying the findings described in manuscript. (XLS)
